# The connexin hemichannel inhibitor D4 produces rapid antidepressant-like effects in mice

**DOI:** 10.1186/s12974-023-02873-z

**Published:** 2023-08-20

**Authors:** Huanhuan Li, Anni Guo, Magdiel Salgado, Juan C. Sáez, Chunyue Geoffrey Lau

**Affiliations:** 1 Department of Neuroscience, City University of Hong Kong, Hong Kong SAR, China; 2 Shenzhen Research Institute, City University of Hong Kong, Shenzhen, China; 3 Instituto de Neurociencias, Centro Interdisciplinario de Neurociencias de Valparaíso, Universidad de Valparaíso, 2381850 Valparaíso, Chile

**Keywords:** Depression, Connexin hemichannels, Glia, Stress, Antidepressant

## Abstract

Depression is a common mood disorder characterized by a range of clinical symptoms, including prolonged low mood and diminished interest. Although many clinical and animal studies have provided significant insights into the pathophysiology of depression, current treatment strategies are not sufficient to manage this disorder. It has been suggested that connexin (Cx)-based hemichannels are candidates for depression intervention by modifying the state of neuroinflammation. In this study, we investigated the antidepressant-like effect of a recently discovered selective Cx hemichannel inhibitor, a small organic molecule called D4. We first showed that D4 reduced hemichannel activity following systemic inflammation after LPS injections. Next, we found that D4 treatment prevented LPS-induced inflammatory response and depressive-like behaviors. These behavioral effects were accompanied by reduced astrocytic activation and hemichannel activity in depressive-like mice induced by repeated low-dose LPS challenges. D4 treatment also reverses depressive-like symptoms in mice subjected to chronic restraint stress (CRS). To test whether D4 broadly affected neural activity, we measured c-Fos expression in depression-related brain regions and found a reduction in c-Fos^+^ cells in different brain regions. D4 significantly normalized CRS-induced hypoactivation in several brain regions, including the hippocampus, entorhinal cortex, and lateral septum. Together, these results indicate that blocking Cx hemichannels using D4 can normalize neuronal activity and reduce depressive-like symptoms in mice by reducing neuroinflammation. Our work provides evidence of the antidepressant-like effect of D4 and supports glial Cx hemichannels as potential therapeutic targets for depression.

**Supplementary Information:**

The online version contains supplementary material available at 10.1186/s12974-023-02873-z.

## Introduction

Major depression is one of the most prevalent mood disorders worldwide. Individuals with depression display a variety of symptoms marked by prolonged depressed mood and diminished interest in activities [1, 2]. Although decades of clinical and basic research have provided significant insights into the pathophysiology of depression, the underlying neural basis remains unclear [3, 4]. Current treatment strategies are not sufficient to alleviate the burden caused by depression. For example, classic antidepressant drugs targeting traditional neurotransmitter systems, like serotonin, have delayed onsets [5]. Novel fast-acting antidepressants, such as ketamine, offer rapid relief from depressive symptoms but face restricted clinical use due to adverse effects, including safety in long-term treatment [6]. The future development of antidepressant medications still requires a better understanding of the neural mechanisms involved in depression and the identification of effective molecular targets.

In the central nervous system, hemichannels formed by connexins (Cxs) or pannexins are essential for neuron-glia communication and maintenance of brain homeostatic balance [7, 8]. In normal conditions, glial Cx-based hemichannels have relatively low activity. Under physiological conditions, neuronal pannexin hemichannels are permeable to small molecules like ATP and are preferentially permeable to Cl^–^ [9, 10]. However, exacerbated neuronal hemichannel activity has been observed in diverse pathological conditions [11]. Under these conditions, hemichannel activity increases and promotes the release of various gliotransmitters, including ATP and glutamate [7]. Hemichannel hyperactivity can alter neuronal excitability and thus promote neuronal excitotoxicity, neuroinflammation, and cell damage in several brain diseases, such as epilepsy and ischemia [12–17]. While recent studies have shown that some hemichannel blockers can provide neuroprotective effects [16, 18], the therapeutic potential of hemichannel regulation has been scarcely investigated.

Downregulation of connexin 43 (Cx43) in the prefrontal cortex of patients with depression [19–21] has been partially validated in an animal model of depression [22]. Interestingly, the blockade of Cx-based channels (hemichannels and gap junction channels) potentiates the effect of antidepressant treatment [23]. Moreover, mounting evidence supports an inhibitory impact of antidepressants on Cx hemichannels. For instance, antidepressant suppresses Cx hemichannel activity induced by LPS in cultured cortical astrocytes [24]. Another study indicated that glutamate release from activated Cx hemichannels could be inhibited with antidepressant drugs [25]. Ketamine, a recently approved fast-acting antidepressant [26], can inhibit hemichannel activity in cortical astrocytes and neurons induced by LPS or pro-inflammatory cytokines [27]. These findings suggest that hemichannels may serve as a promising target for antidepressant treatments. However, although recent clinical and preclinical findings suggest a role for Cx hemichannels in depression [28], in vivo results supporting the role of hemichannels in alleviating depressive-like symptoms are scarce.

In a recent study, we screened and identified a novel Cx hemichannel inhibitor, which is a small organic molecule called D4 that has been shown to be effective in reducing the atrophy in pathological muscular dysfunction [29] as well as alleviating neuroinflammation in temporal lobe epilepsy [16]. Here, we hypothesize that blocking Cx hemichannels can alleviate neuroinflammatory and depressive-like symptoms. Using a combination of dye uptake experiments, immunofluorescent staining of astrocytes and microglia, immediate early gene c-Fos expression, and behavioral analysis, we investigated the antidepressant-like efficacy of D4. We found that blocking mainly Cx hemichannels with D4 produces antidepressant-like effects by suppressing neuroinflammation.

## Methods

### Animals

Adult C57BL/6 mice (2–4 months old) were used in this study. Male mice were used throughout this study except for data presented in Figs. 2 and 4, where both male and female mice were used. Mice were acclimated to the Laboratory Animal Research Unit (LARU) at the City University of Hong Kong. Mice were group housed in a 12 h dark/light cycle (light on/off at 08:00/20:00). Food and water were given ad libitum. All animal procedures were approved by the LARU and were conducted in accordance with guidelines from the Animal Research Ethics Sub-Committees of the City University of Hong Kong and the Department of Health of the Government of the Hong Kong Special Administrative Region.

### Drugs

The chemical compound D4 [16, 29], (R)-2-(4-chlorophenyl)-2-oxo-1-phenylethyl quinoline-2-carboxylate (Molecular weight 401.85 g/mol), was synthesized using a series of chiral synthetic methods (Qianyan Shenzhen Pharmatech). For in vivo experiments, D4 was dissolved in 100% ethanol as stock (20 mg/mL) and diluted with sterile 0.9% saline to the desired dosage right before use. For adult mice weighed about 30 g, either the vehicle (10% ethanol in sterile 0.9% saline) or the D4 suspension (0.5 mg/kg or 5 mg/kg body weight, i.e., 15 µl or 150 µl D4 suspension, respectively) was administered orally (p.o.) under gentle restraint without anesthesia. For ex vivo dye loading experiments, the D4 stock solution (20 mg/mL) was diluted with artificial cerebrospinal fluid (ACSF) to the desired dosage before use. For the dye loading experiments after acute lipopolysaccharides (LPS) challenges, three different doses of D4 solution were used (100 nM D4 containing 0.0002% EtOH in ACSF, 1 µM D4 containing 0.002% EtOH in ACSF, and 10 µM D4 containing 0.02% EtOH in ACSF). Mice were randomly grouped in these experiments.

### CBF dye loading

Hemichannel opening in brain slices was assessed by CBF (5(6)-Carboxyfluorescein, 21877, Sigma-Aldrich) dye uptake as previously published [30]. Briefly, acute coronal hippocampal slices (300 µm) were prepared in ice-cold oxygenated (95% O_2_ and 5% CO_2_) ACSF containing (in mM) 119 NaCl, 2.5 KCl, 2.5 CaCl_2_, 1.3 MgSO_4_, 1 NaH_2_PO_4_, 26.2 NaHCO_3_, and 22 glucose. For the dye loading experiments after acute LPS challenges, acute slices were transferred to ACSF (vehicle) or D4 and incubated for 20 min. CBF (100 µM) was added to ACSF saturated with 95% O_2_ and 5% CO_2_ at room temperature. After pre-treatment, slices were then transferred to ACSF containing CBF for 20 min. For the dye loading experiments after repeated LPS challenges or CRS, acute slices were incubated with ACSF saturated with 95% O_2_ and 5% CO_2_ at room temperature for 20 min. Then, incubated slices were transferred to ACSF containing CBF (100 µM) saturated with 95% O_2_ and 5% CO_2_ at room temperature for 20 min. Slices were washed three times with ACSF and fixed with 4% paraformaldehyde at 4 °C overnight. Slices were cryoprotected in 30% sucrose for 36 h before being fast frozen, followed by cryosectioning. Brain sections (15 µm thick) were prepared with a cryostat (HM525 NX, Thermo Fisher Scientific) for immunostaining and confocal microscopy. Fluorescence images were quantified using ImageJ (National Institutes of Health). Briefly, background subtraction was applied to the whole image to reduce non-specific fluorescence signals of raw images. Quantitative analysis was performed on thresholded images to analyze the area or integrated density (IntDen) of positive signals. For a given marker, the threshold value and size for particle analysis were held constant for each set of experiments. To analyze CBF uptake in different neural cells, thresholded images of the channel containing either glial fibrillary acidic protein (GFAP), allograft inflammatory factor 1 (Iba1), or Nissl were first used to extract regions of interest (ROIs) representing astrocyte, microglia, or neuronal somata, respectively. Then, CBF uptake in each cell type was measured by quantifying CBF-positive areas of the selected ROIs.

### Mouse models of depression

Inflammation-based mouse model of depression: Systemic inflammation activated by LPS (L2880, Sigma-Aldrich) can induce depressive-like behaviors in mice. The LPS-induced depression model was conducted as previously described [31]. LPS was dissolved in sterile 0.9% saline. Mice were intraperitoneally (i.p.) injected with LPS or saline at a 0.75 mg/kg dose between 10:00 to 12:00 daily for 1 week. Body weight was measured before each injection. Behavioral assays were performed 24 h after the last injection.

Stress-induced mouse model of depression: The CRS-induced depression model was used as previously described [32]. Mice were restrained in the ventilated 50 mL Falcon tubes (with sixteen 3.5 mm air vents at the wall and one 2.0 mm air vent at the nasal end of the tube) for 6 h per day during the light cycle. Mice were able to move their head and body but could not escape. During the restraint, animals had no access to food/water or social interaction. As a control, a separate cohort of mice was subjected to food and water deprivation for 6 h. Body weight was measured once a week. Behavioral assays were performed 24 h after the last restraint.

### Behavioral assays

All behavioral tests were performed during the light cycle. Mice were habituated to the behavior room for at least 1 h before testing. ToxTrac software was used to analyze the behavioral videotape offline [33]. The behavior box was cleaned with 70% ethanol after each trial to eliminate any olfactory cues.

Open field test (OFT): Mice were randomly placed in one corner of the open-field arena (50 cm × 50 cm × 40 cm, Length × Width × Height) with light (400 lx) and were allowed to explore freely for 10 min. A top camera above the arena was used to record mouse movement. The total distance and time spent in the center of the arena (25 cm × 25 cm) were analyzed using Toxtrac software by tracking the mouse centroid. The total distance indicated the locomotor and exploratory activities of mice. Less time spent in the center suggests anxiety-like behavior [34].

Tail suspension test (TST): Mice were suspended by their tails and secured with tape for 6 min in a behavior box with dim light (100 lx). Each mouse was separated into its three-walled area without visual interaction. A 3D-printed white hollow tube was used to prevent climbing during the test. A side camera was placed in the behavior box to record the mouse's movement. The mouse speed was measured by tracking the mouse centroid using Toxtrac software. Total immobility time (speed ≤ 0.5 cm/s) or struggling time (speed > 5 cm/s) was counted during the last 4 min. Increased immobility or decreased struggling suggests depressive-like behavior [35].

Forced swim test (FST): Mice were introduced to a cylindrical container (22 cm inner diameter, 20 cm depth) filled with 10 cm-deep tap water (22–24 °C) and allowed to swim for 6 min. A top camera above the behavior box was used to record the mouse's movement. Mouse speed was analyzed by tracking the mouse centroid using Toxtrac software. Total immobility time (speed ≤ 1.25 cm/s) was counted during the last 4 min. An increase in immobility suggests depressive-like behavior [36].

Sucrose preference test (SPT): Mice were habituated for 48 h to 1% sucrose water, followed by 24 h of water deprivation. During the test day, mice were placed in the behavior box with a pre-weighed bottle filled with 1% sucrose or plain water for 1 h to determine their preference for 1% sucrose or water. Bottles were weighed after the test. Sucrose preference was expressed as (sucrose intake)/(sucrose intake + water intake) × 100. Reduced sucrose preference suggests anhedonia [37].

### Immunofluorescence and analysis

After behavioral tests, animals were deeply anesthetized with a 10% ketamine/1% xylazine mixture and were then perfused with 4% paraformaldehyde (PFA) in 1X phosphate-buffered saline (PBS) at room temperature for 10 min. The whole brain was post-fixed overnight in 4% PFA at 4 °C followed by cryoprotection in 30% sucrose in PBS at 4 °C for 72 h. Brain sections (30 µm thick) were prepared with a cryostat (HM525 NX, Thermo Fisher Scientific). For immunohistochemistry, the sections were washed three times with 1X PBS for 10 min each, followed by 10% normal goat serum in 1X PBS. Preparations with primary antibodies (rabbit anti-GFAP, 1:500, Z0334, Dako; rabbit anti-Iba1, 1:500, 019-19741, Wako; rabbit anti-c-Fos, 1:500, ab190289, Abcam) were applied for overnight at 4 °C. Next, sections were incubated with the secondary antibody (Jackson ImmunoResearch) with 4′,6-diamidino-2-phenylindole (DAPI, 28718-90-3, Santa Cruz Biotechnology) and/or Nissl stain (NeuroTrace 640/660, N21483, Thermo Fisher Scientific) at room temperature for 2 h in darkness. After washing with 1X PBS, sections were mounted (Antifade mounting medium, H-1000, Vector Laboratories). The stained sections were stored in the dark box at 4 °C before imaging. Images were visualized by epifluorescence (Nikon Eclipse Ni-E upright fluorescence microscope, Nikon) or confocal microscopy (LSM880, ZEISS). Fluorescence images were quantified using ImageJ (National Institutes of Health) as described above. Briefly, the raw images were processed with background subtraction. To quantify the number of positive cells, the threshold value and size for particle analysis were manually adjusted for each marker.

### Cytokine analysis

Mouse blood samples and hippocampi were harvested 4 h after the last LPS injection and drug treatment. To collect plasma, blood was collected into tubes containing ethylenediaminetetraacetic acid (EDTA) and chilled on ice before centrifugation. Plasma was separated by refrigerated centrifugation at 2000×*g* for 20 min (4 °C) within 1 h of collection. After centrifugation, the plasma was immediately extracted and then frozen at − 20 °C before use. For protein extraction, hippocampi were dissected on ice and rinsed three times with ice-cold 1X PBS. Then, hippocampi were lysed in the radioimmunoprecipitation assay (RIPA) buffer with a protease inhibitor cocktail (1:200), sodium pyrophosphate (1 mM), and 20 mM sodium fluoride (Sigma-Aldrich). The hippocampal lysates were centrifuged at 12,000 rpm for 15 min at 4 °C to collect the supernatant. Protein concentrations were determined with DC protein assay kit (5000111, Bio-Rad). Plasma and hippocampal interleukin-1β (IL-1β) protein levels were measured by an enzyme-linked immunosorbent assay (ELISA) (EK0394, Boster Biological Technology) according to the manufacturer’s instructions by fluorescence measurement at 450 nm (Synergy H1 Microplate Reader, BioTek Instruments). The level of IL-1β was expressed as fold changes compared to the saline/vehicle control group average.

### Statistics

Statistical analysis was performed using SPSS 25 (International Business Machines Corporation, IBM). For comparisons between data with normal distributions and equal variances, one-way analysis of variance (ANOVA) was used to check differences between multiple groups. Student’s t-test was performed to test differences between two groups. A nonparametric test was used when data did not pass tests for normality and equal variance. Unless otherwise specified, data are shown as mean ± s.e.m. Thresholds for significance were indicated as *p < 0.05, **p < 0.01, and ***p < 0.001. The details of statistical tests and results are shown in the figure legends.

## Results

### D4 inhibits hemichannel activity induced by LPS-mediated neuroinflammation

**Fig. 1:**
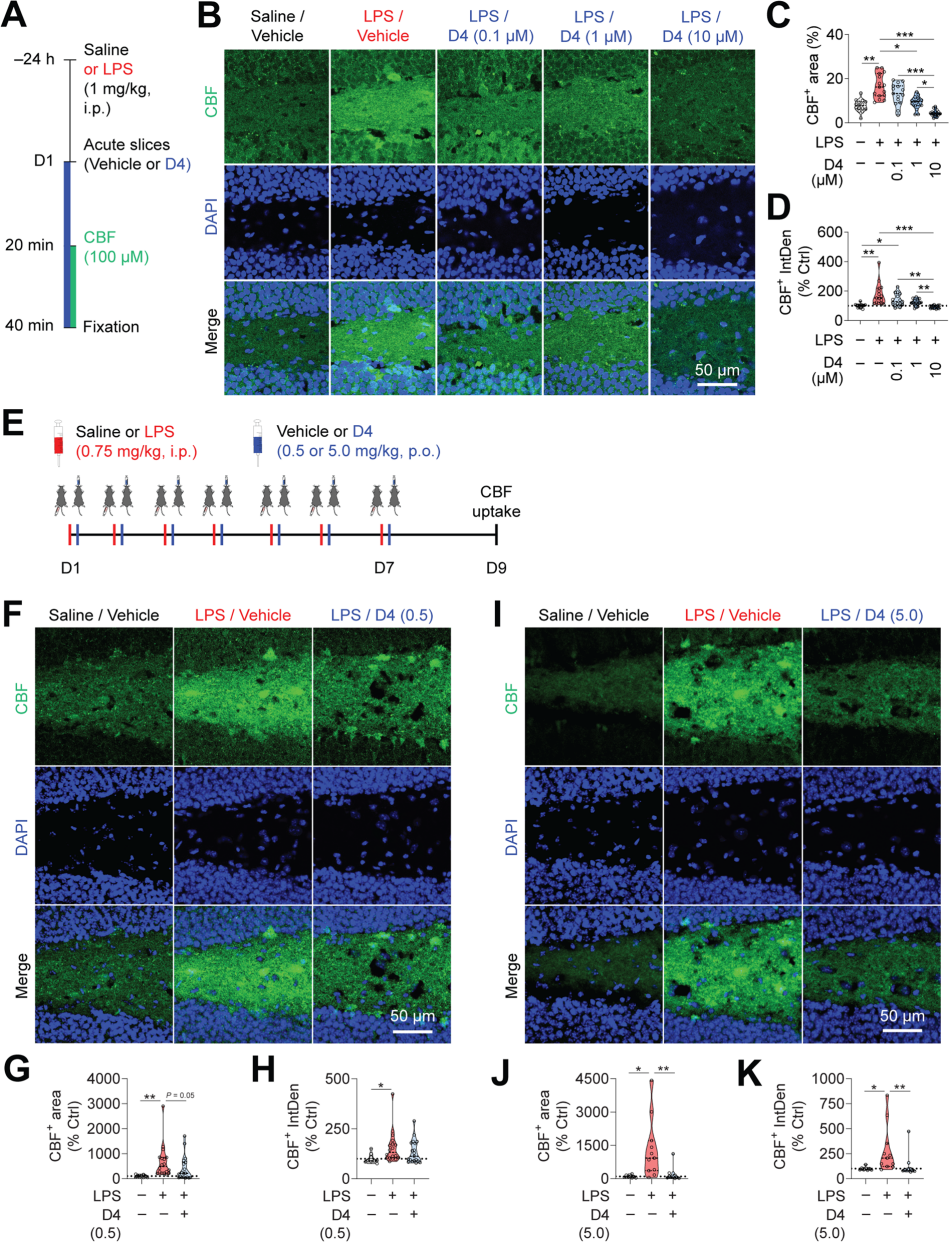
D4 inhibits LPS-induced hemichannel activity. **A** Experimental design to examine CBF uptake after acute systemic bacterial lipopolysaccharide (LPS) injection. **B** Representative images showing the CBF uptake in the acute hippocampal dentate gyrus (DG). CBF, green; DAPI, blue. Scale bar: 50 µm. **C** Quantification of CBF^+^ area in the stained DG sections. **D** Normalized CBF^+^ integrated density (IntDen) in the DG sections of saline/vehicle control (Ctrl), LPS/vehicle, LPS/D4 (0.1 µM), LPS/D4 (1 µM), and LPS/D4 (10 µM). The concentration of ethanol in D4 solution is no more than 0.02% v/v. n = 15 DG sections from 5 mice for all groups. **E** Protocol to measure CBF uptake after repeated systemic low-dose LPS injections. **F** Representative images showing the CBF uptake in the DG of mice received vehicle (Veh) or low-dose D4 (0.5 mg/kg, p.o.). CBF, green; DAPI, blue. Scale bar: 50 µm. **G** Violin plot of normalized CBF^+^ area in the DG of LPS/vehicle and LPS/D4 (0.5 mg/kg, p.o.) treated mice. **H** Violin plot of normalized CBF^+^ integrated density in the DG. Saline/vehicle, white, n = 9 DG sections from 3 mice; LPS/vehicle, red, n = 18 DG sections from 7 mice; LPS/D4 (0.5 mg/kg, p.o.), blue, n = 19 DG sections from 7 mice. **I** Representative images showing the CBF uptake in the DG of mice received vehicle (Veh) or higher dose D4 (5.0 mg/kg, p.o.). **J** Violin plot of normalized CBF^+^ area in the DG. **K** Violin plot of normalized CBF^+^ integrated density in the DG of LPS/vehicle and LPS/D4 (5.0 mg/kg, p.o.) treated mice. Saline/vehicle, white, n = 8 DG sections from 3 mice; LPS/vehicle, red, n = 11 DG sections from 5 mice; LPS/D4 (5.0 mg/kg, p.o.), blue, n = 10 DG sections from 4 mice. The open circle shows the value of each DG. Kruskal–Wallis test with *Bonferroni* correction (**C**, **D**, **G**, **H**, **J**, **K**). *p < 0.05, **p < 0.01, ***p < 0.001

Hemichannel opening can be triggered by multiple processes, such as increases in intracellular Ca^2+^ concentration, acute and chronic stress, and inflammatory activation [38–41]. Previously, our results suggested that D4 blocks Cx26, Cx30, and Cx43 hemichannels [16, 29]. In cultured HeLa cells, we also found D4 did not affect the activity of other non-selective channels that share structural, functional, and pharmacologic sensitive similarity with Cx [42], such as the transient receptor potential cation channel subfamily V member 2 (TRPV2) channels (Additional file 1: Fig. S1), purinergic ionotropic P2X7 receptor (P2X_7_R) channels (Additional file 2: Fig. S2), and calcium homeostasis modulator 1 (CALHM1) channels (Additional file 3: Fig. S3). These channels were shown to participate in brain dysfunctions and are permeable to Ca^2+^, a critical element in neuroinflammatory responses [43]. Given that small molecules can permeate through Cx-based hemichannels [7], we next used the fluorescent dye 5(6)-carboxyfluorescein (CBF) to measure hemichannel activity in the brain [30, 44]. To create a model of depression in mice, we intraperitoneally injected a single dose of lipopolysaccharide (LPS, 1 mg/kg, i.p.) into adult mice to induce neuroinflammation. The hippocampus and its subregions, such as the dentate gyrus (DG), have been implicated in depression [4]. Therefore, twenty-four hours later, we made acute hippocampal slices, measured CBF accumulation in the DG, and tested whether D4 in vitro could prevent hemichannel-mediated dye uptake (Fig. 1A). We found that cell membrane permeability mediated by hemichannel was very low in the DG from the saline/vehicle control mice (CBF^+^ area, 7.7 ± 0.7%; CBF^+^ integrated density or IntDen, 100.0 ± 3.3%). In contrast, LPS increased hemichannel activity, as evidenced by robust increases in CBF uptake in the DG from the LPS/vehicle mice (CBF^+^ area, 16.6 ± 1.4%; CBF^+^ IntDen, 178.3 ± 19.6%). In acute slices incubated with D4, a low dose of D4 (0.1 µM) did not affect LPS-induced increase in CBF uptake (D4 at 0.1 µM: CBF^+^ area, 13.0 ± 1.3%; CBF^+^ IntDen, 140.0 ± 11.0%). A medium dose of D4 (1 µM) significantly reduced CBF uptake induced by LPS (D4 at 1 µM: CBF^+^ area, 9.0 ± 7.6%; CBF^+^ IntDen, 122.6 ± 5.7%). The higher dose of D4 (10 µM) almost completely abolished the increase in LPS-mediated CBF uptake in the DG (D4 at 10 µM: CBF^+^ area, 4.3 ± 3.8%; CBF^+^ IntDen, 92.4 ± 2.6%) (Fig. 1B–D). These results suggest that D4 can concentration-dependently suppress hemichannel activity caused by pro-inflammatory conditions.

We next asked whether in vivo chronic treatment with D4 can inhibit Cx hemichannel activity induced by repeated low-dose LPS injections. Mice were subjected to intraperitoneal injection with saline or LPS (0.75 mg/kg) followed by D4 oral administration (0.5 mg/kg or 5.0 mg/kg) daily for 1 week. After one day of recovery from the injections, we assessed CBF uptake in acute hippocampal slices (Fig. 1E). In vivo application of D4 produced a dose-dependent effect. While a low dose of D4 (0.5 mg/kg) resulted in some reduction in LPS-induced CBF uptake (area: saline/vehicle, 100.0 ± 17.8%, LPS/vehicle, 671.3 ± 154.4%, LPS/D4 at 0.5 mg/kg, 394.6 ± 110.9%; IntDen, saline/vehicle, 100.0 ± 7.9%, LPS/vehicle, 156.9 ± 18.9%, LPS/D4 at 0.5 mg/kg, 134.6 ± 13.2%) (Fig. 1F–H), the higher dose of D4 (5.0 mg/kg) strongly inhibited CBF uptake (area: saline/vehicle, 100.0 ± 22.2%, LPS/vehicle, 1313.4 ± 402.1%, LPS/D4 at 5.0 mg/kg, 191.2 ± 105.6%; IntDen, saline/vehicle, 100.0 ± 6.7%, LPS/vehicle, 284.9 ± 71.9%, LPS/D4 at 5.0 mg/kg, 129.2 ± 39.0%) compared to the saline/vehicle control (Fig. 1I–K). Since 5.0 mg/kg D4 had a stronger and more significant effect on CBF uptake, this dose was used in the following behavioral experiments. These results show that D4 can inhibit hemichannel activity both in vitro and in vivo.

### D4 prevents LPS-induced depressive-like behaviors

**Fig. 2:**
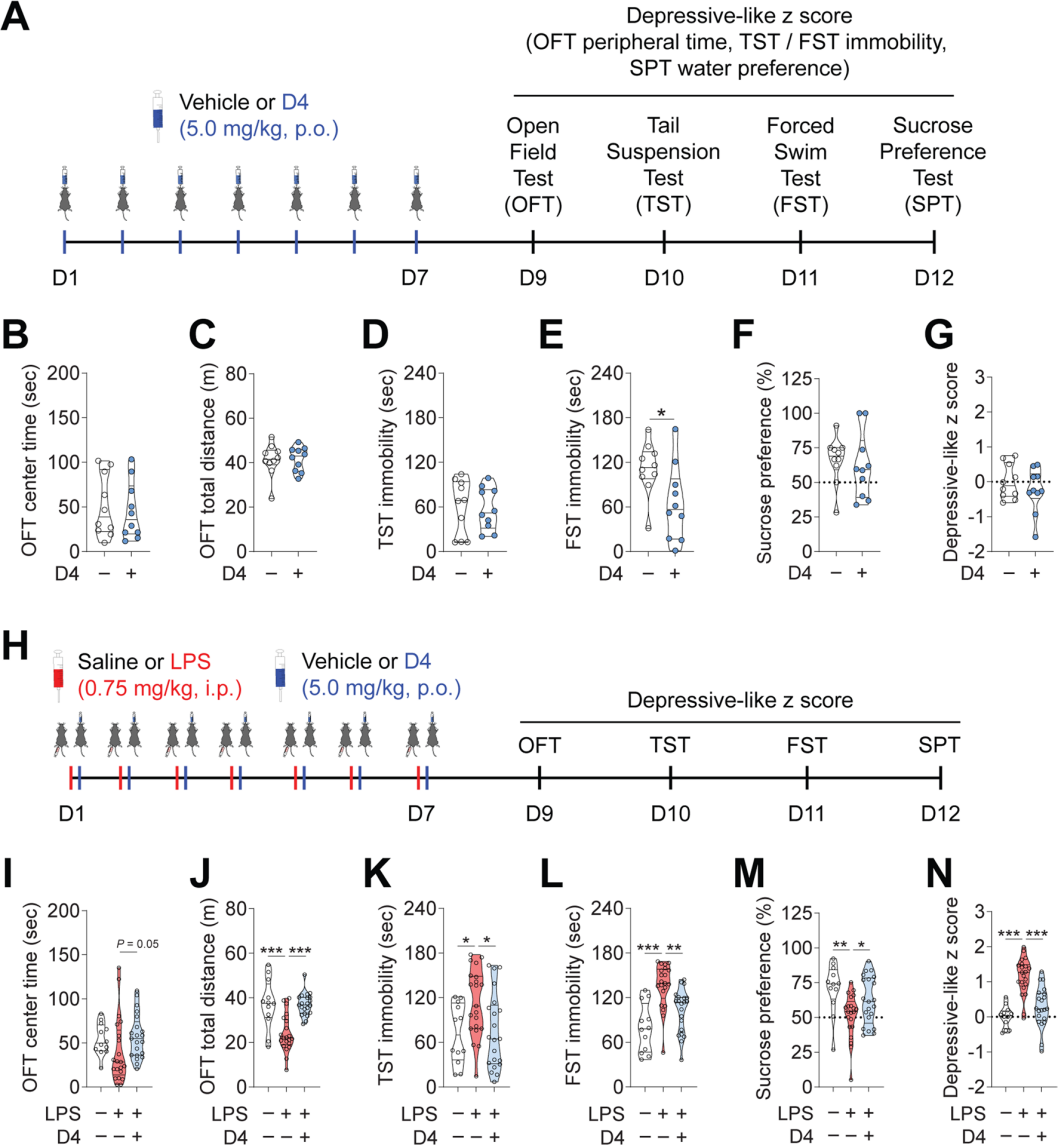
D4 prevents depressive-like behaviors induced by repeated low-dose LPS challenges. **A** Schematic of drug treatment and behavioral testing. **B** Center time and **C** total distance in the OFT. **D** Time immobile in the TST. **E** Time immobile in the FST. **F** Sucrose preference in the SPT. **G** Depressive-like z score of the unstressed mice treated with vehicle or D4. Ctrl/vehicle, n = 10 mice, 5 male and 5 female; Ctrl/D4, n = 10 mice, 5 male and 5 female. **H** Timeline of LPS injection, drug treatment, and behavioral testing. **I** Center time and **J** total distance in the OFT for saline/vehicle control, LPS/vehicle treated, and LPS/D4 treated mice. **K** Time immobile in the TST. **L** Time immobile in the FST. **M** Sucrose preference in the SPT. **N** Depressive-like z score of saline/vehicle control, LPS/vehicle, and LPS/D4 mice. Saline/vehicle, n = 12 mice, 7 male and 5 female; LPS/vehicle, n = 21 mice, 15 male and 6 female; LPS/D4, n = 21 mice, 12 male and 9 female. The filled dot indicates the value of each mouse. Data are mean ± s.e.m. Unpaired Student's t-test (**B–G**). One-way ANOVA followed by Fisher’s Least Significant Difference (LSD) post hoc test (**J**, **K**, **M**, **N**). Kruskal–Wallis test with *Bonferroni* correction (**I**, **L**). *p < 0.05, ***p < 0.001

Numerous studies have shown that neuroinflammation plays a pivotal role in the regulation of depression [45–48]. Both acute and repeated systemic LPS challenges have been widely used as inflammation-based models of depression [49, 50]. Given that D4 inhibited hemichannel activity induced by LPS (Fig. 1), we then sought to determine the behavioral outcome of D4. First, we assessed the behavioral effects of D4 (5 mg/kg, p.o.) following 1 week of oral administration in unstressed mice (Fig. 2A). We found that D4-treated mice showed no differences in center time (Vehicle, 51.0 ± 11.0 s; D4, 45.3 ± 10.2 s) or total distance (Vehicle, 41.2 ± 2.3 m; D4, 41.6 ± 1.7 m) in the open field test (OFT) compared to vehicle-treated mice (Fig. 2B, C). This suggests that D4 does not affect thigmotaxis or locomotor activity in healthy control mice. In unstressed mice, although D4 did not alter immobility in the tail suspension test (TST; Vehicle, 60.1 ± 11.6 s; D4, 57.2 ± 9.0 s), it moderately reduced immobility (by 41.7%) in the forced swim test (FST; Vehicle, 111.4 ± 11.2 s; D4, 65.0 ± 16.0 s) (Fig. 2D, E). In the sucrose preference test (SPT), mice receiving D4 displayed similar sucrose preference compared to their vehicle control mice (Vehicle, 66.0 ± 5.3%; D4, 61.7 ± 7.5%) (Fig. 2F).

Next, we tested whether pharmacological inhibition of hemichannels with D4 could affect depressive-like behaviors induced by repeated low-dose (0.75 mg/kg) LPS challenges (Fig. 2H). In the OFT, LPS-injected mice showed a small decrease in center time, which was reversed by chronic D4 application (Saline/vehicle, 54.1 ± 5.2 s, LPS/vehicle, 40.8 ± 8.2 s; LPS/D4, 57.3 ± 5.5 s) (Fig. 2I). Mice that received LPS exhibited a significant reduction in OFT total distance. Treatment with D4 restored the total distance travelled in the OFT arena (Saline/vehicle, 37.2 ± 3.2 m; LPS/vehicle, 24.0 ± 1.8 m; LPS/D4, 37.0 ± 1.1 m) (Fig. 2J). In the TST, the immobility time of LPS-injected mice was significantly increased compared to that of saline/vehicle control mice. LPS mice administrated with D4 exhibited significant decreases in TST immobility (Saline/vehicle, 71.4 ± 11.6 s; LPS/vehicle, 113.3 ± 9.9 s; LPS/D4, 75.0 ± 11.1 s) (Fig. 2K). In LPS-injected mice, the D4-induced rescue in immobility was also observed in the FST (Saline/vehicle, 79.1 ± 9.4 s; LPS/vehicle, 136.1 ± 6.5 s; LPS/D4, 102.4 ± 6.3 s) (Fig. 2L). We next performed the SPT to measure anhedonia, a core clinical symptom of depression. D4 improved LPS-mediated reduction in the sucrose preference in the LPS-injected mice (Saline/vehicle, 70.9 ± 5.2%; LPS/vehicle, 51.3 ± 3.5%; LPS/D4, 62.1 ± 3.7%) (Fig. 2M).

To more robustly evaluate depressive-like symptoms in mice, we used Z normalization to evaluate the behavioral performance in four tests of OFT, TST, FST, and SPT [51]. Overall, D4 did not change depressive-like z scores in unstressed mice (Vehicle, 0.00 ± 0.16; D4, − 0.24 ± 0.20) (Fig. 2A, G). Repeated LPS challenge induced significantly higher depressive-like z scores in mice, suggesting the expression of depressive-like behavior. This behavioral effect induced by LPS was reversed by D4 (Saline/vehicle, 0.00 ± 0.09; LPS/vehicle, 1.15 ± 0.11; LPS/D4, 0.28 ± 0.12) (Fig. 2N). Taken together, these results demonstrate that blocking preferentially Cx hemichannels with D4 exerts antidepressant-like effects in mice.

### D4 suppresses LPS-induced astrocytic activation and hemichannel activity

**Fig. 3:**
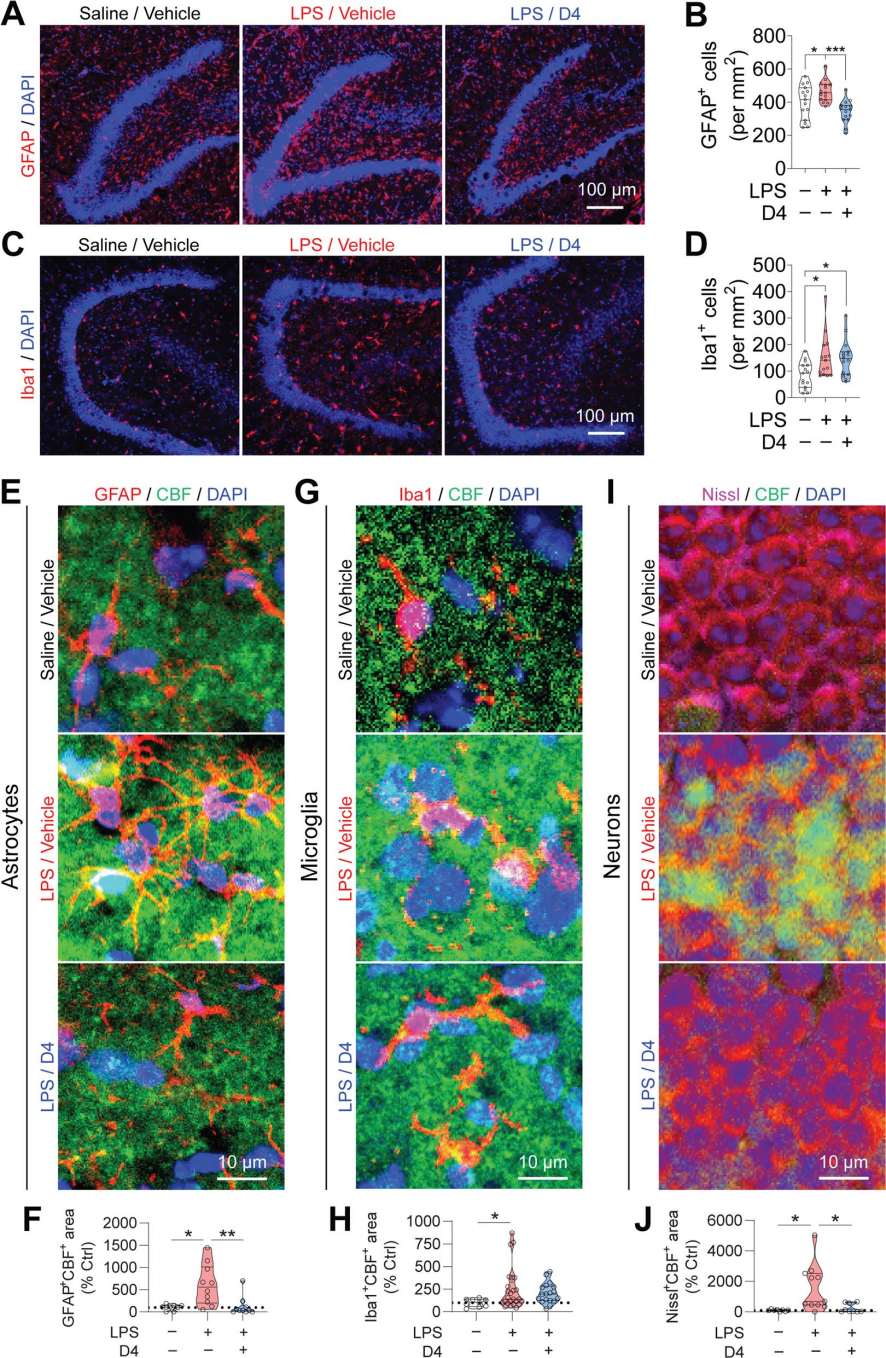
D4 reduces astrocytic proliferation and hemichannel activity induced by repeated low-dose LPS injections. **A** Immunostaining for astrocyte (GFAP) in the hippocampus. GFAP, red; DAPI, blue. Scale bar, 100 µm. **B** Quantification of GFAP^+^ astrocytes in the hippocampus. n = 15 hippocampal sections from 5 mice for all groups. **C** Immunostaining for microglia (Iba1) in the hippocampus. Iba1, red; DAPI, blue. Scale bar, 100 µm. **D** Quantification of Iba1^+^ cells in the hippocampus. n = 15 hippocampal sections from 5 mice for all groups. **E** Representative images showing astrocytes (GFAP) and CBF uptake in the hilus of the hippocampal DG from the LPS-injected mice. GFAP, red; CBF, green; DAPI, blue. Scale bar, 10 µm. **F** Quantification of GFAP^+^CBF^+^ area in the stained DG sections. Saline/vehicle, n = 8 sections from 3 mice; LPS/vehicle, n = 10 DG sections from 5 mice; LPS/D4, n = 10 DG sections from 4 mice. **G** Representative images showing microglia (Iba1) and CBF uptake in the hilus of the hippocampal DG the LPS-injected mice. Iba1, red; CBF, green; DAPI, blue. Scale bar, 10 µm. **H** Quantification of Iba1^+^CBF^+^ area in the stained DG sections. Saline/vehicle, n = 9 sections from 3 mice; LPS/vehicle, n = 25 DG sections from 9 mice; LPS/D4, n = 18 DG sections from 6 mice. **I** Representative images showing neurons (Nissl) and CBF uptake in the granule cell layer of the hippocampal DG the LPS-injected mice. Nissl, magenta; CBF, green; DAPI, blue. Scale bar, 10 µm. **J** Quantification of Nissl^+^CBF^+^ area in the stained DG sections. Saline/vehicle, n = 8 sections from 3 mice; LPS/vehicle, n = 11 DG sections from 5 mice; LPS/D4, n = 9 DG sections from 4 mice. Saline/vehicle (Ctrl), white; LPS/vehicle, red; LPS/D4, blue. The open circle shows the value from each DG. One-way ANOVA followed by LSD post hoc test (**B**). Kruskal–Wallis test with *Bonferroni* correction (**D**, **F**, **H**, **J**). *p < 0.05, **p < 0.01, ***p < 0.001

Reactive glial cells are hallmarks of neuroinflammation and have been implicated in the pathophysiology of depression [52–55]. Having demonstrated the antidepressant-like properties of D4, we first investigated the effect of D4 on LPS-mediated inflammation. We injected adult mice intraperitoneally with a low dose of LPS (0.75 mg/kg, i.p.) or saline (control) daily for 1 week. Mice were orally fed with either D4 (5 mg/kg, p.o.) or vehicle after each LPS injection. Four hours after the last LPS injection, plasma and hippocampi were extracted for ELISA assay. We found that repeated LPS injections significantly increased pro-inflammatory cytokine IL-1β levels in the plasma and hippocampi. Notably, D4 significantly attenuated LPS-induced increases in the plasma and hippocampal IL-1β levels (Additional file 4: Fig. S4). We next tested whether D4 can affect reactive glia in LPS-induced depressive-like mice. After LPS injections and D4 treatment, mice were sacrificed after the last behavioral testing. We used GFAP as a marker to label astrocytes. Injections of LPS significantly increased astrocyte density in the hippocampus. Importantly, administration of D4 significantly decreased the number of GFAP^+^ astrocytes in the hippocampus (Saline/vehicle, 400.6 ± 26.3; LPS/vehicle, 464.6 ± 16.7; LPS/D4, 343.5 ± 17.5) (Fig. 3A, B). However, LPS-mediated hippocampal reactive microgliosis (Iba1^+^ microglia) was unaffected by D4 (Saline/vehicle, 85.2 ± 12.8; LPS/vehicle, 152.9 ± 21.0; LPS/D4, 149.2 ± 17.8) (Fig. 3C, D).

To directly interrogate whether D4 has a stronger impact on the hemichannel activity of astrocytes or microglia, we co-stained for GFAP or Iba1 after CBF dye loading in LPS mice. In the DG, D4 drastically suppressed LPS-induced CBF uptake in GFAP-positive astrocytes compared to saline/vehicle control (Saline/vehicle, 100.0 ± 23.9%; LPS/vehicle, 609.6 ± 145.6%; LPS/D4, 122.2 ± 67.9%), suggesting that D4 inhibits dye uptake in astrocytes triggered by LPS (Fig. 3E, F). By contrast, D4 only had a minimal effect on CBF uptake evoked by LPS in the Iba1-positive microglia (Saline/vehicle, 100.0 ± 14.4%; LPS/vehicle, 246.1 ± 46.0%; LPS/D4, 207.5 ± 26.3%) (Fig. 3G, H). In addition, we found that D4 significantly reduced CBF uptake induced by LPS in Nissl-positive neurons in the hippocampal DG (Saline/vehicle, 100.0 ± 20.6%; LPS/vehicle, 1566.0 ± 461.3%; LPS/D4, 236.5 ± 97.8%) (Fig. 3I, J).

Together, these data suggest that D4 can preferentially block astrocytic Cx hemichannels, and that LPS-induced increases in membrane permeability of microglia might be mediated by other pathways not inhibited by D4, such as pannexin1 hemichannels [7].

### D4 improves depressive-like behaviors in mice subjected to chronic restraint stress

**Fig. 4:**
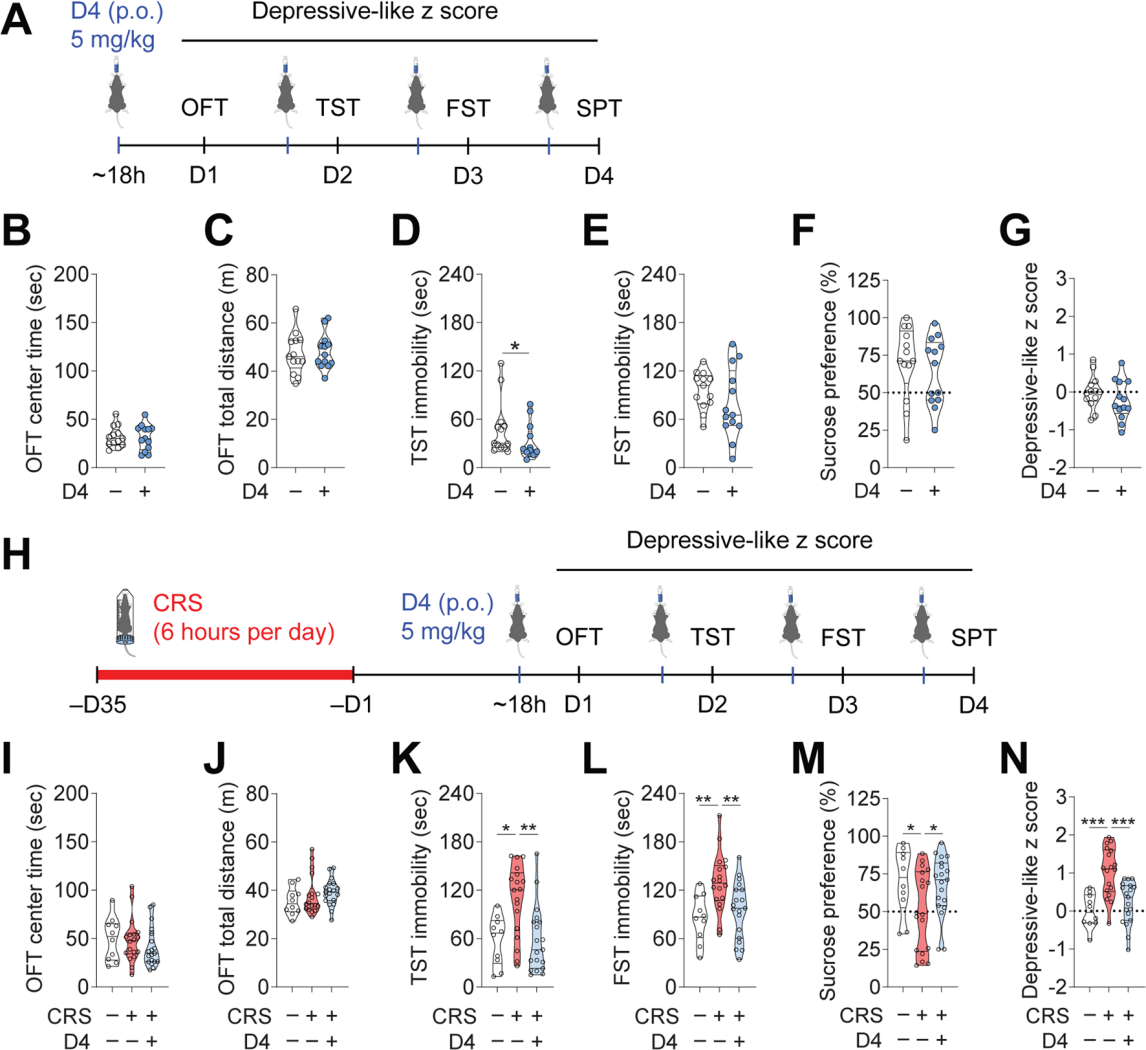
D4 improves depressive-like behaviors induced by chronic restraint stress (CRS). **A** Schematic of drug treatment and behavioral testing. **B** Center time and (**C**) total distance in the OFT. **D** Time immobile in the TST. **E** Time immobile in the FST. **F** Sucrose preference in the SPT. **G** Depressive-like z score of the unstressed mice treated with vehicle or D4. n = 13 mice (5 male and 8 female) for each group. **H** Schematic of the design of CRS, drug treatment, and behavioral testing. **I** Center time and **J** total distance in the OFT. **K** Time immobile in the TST. **L** Time immobile in the FST. **M** Sucrose preference in the SPT. **N** Normalized depressive-like z score of control/vehicle, CRS/vehicle, and CRS/D4 treated mice. Control/vehicle, n = 10 mice, 5 male and 5 female; CRS/vehicle, n = 18 mice, 9 male and 9 female; CRS/D4, n = 19 mice, 10 male and 9 female. The filled dot indicates the value of each mouse. Data are mean ± s.e.m. Unpaired Student's t-test (**B**, **C**, **E**–**G**). Mann–Whitney *U* test (**D**). One-way ANOVA followed by LSD post hoc test (**L–N**). Kruskal–Wallis test with *Bonferroni* correction (**I–K**). *p < 0.05, **p < 0.01, ***p < 0.001

To further validate the antidepressant-like potentials of D4, we asked whether D4 could reverse behavioral changes induced by CRS, a stress paradigm used for developing depressive-like phenotypes in mice [56, 57]. We first assessed the behavioral effects of D4 on unstressed control mice using an alternative testing protocol. Mice were orally fed with either a vehicle or D4 (5 mg/kg) 16–18 h before each behavioral test (Fig. 4A). We found no differences in the OFT center time (Vehicle, 31.8 ± 3.0 s; D4, 31.2 ± 3.7 s) and total distance (Vehicle, 47.4 ± 2.4 m; D4, 48.2 ± 2.0 m) between vehicle and D4 treated mice (Fig. 4B, C). The latter could confirm that D4 had no detectable effect on anxiety-like behavior and locomotor activity in healthy control mice. In the TST, D4 induced a significant reduction in immobility (by 34.5%) at 18 h after treatment (Vehicle, 48.1 ± 9.5 s; D4, 31.5 ± 6.1 s) (Fig. 4D). D4-treated mice showed a small but insignificant decrease (by 17.8%) in FST immobility (Vehicle, 96.1 ± 6.5 s; D4, 79.1 ± 12.6 s) (Fig. 4E). In the SPT, treatment with D4 did not change sucrose preference in unstressed mice (Vehicle, 70.3 ± 6.7%; D4, 64.0 ± 6.2%) (Fig. 4F).

Next, we tested whether D4 could affect depressive-like behaviors in the mice subject to CRS. In the CRS-induced mouse model of depression, mice were exposed to 6-h restraint stress daily for 5 weeks. After the CRS paradigm, mice were orally treated with either vehicle or D4 (5 mg/kg, p.o.) eighteen hours before each behavioral test (Fig. 4H). In the OFT, we observed no significant differences in center time (Control/vehicle, 49.8 ± 7.1 s; CRS/vehicle, 48.2 ± 5.4 s; CRS/D4, 41.1 ± 4.7 s) or total distance (Control/vehicle, 35.4 ± 1.8 m; CRS/vehicle, 38.0 ± 1.9 m; CRS/D4, 39.2 ± 1.2 m) across groups following only a single dose of D4 (Fig. 4I, J). We then measured behavioral despair in the TST and FST. D4 significantly reduced LPS-induced increases in the TST immobility in CRS-exposed mice (Control/vehicle, 57.8 ± 9.6 s; CRS/vehicle, 108.4 ± 10.4; CRS/D4, 58.5 ± 9.4 s) (Fig. 4K). Again, D4 induced a significant decrease in FST immobility time in CRS-exposed mice 18 h after treatment (Control/vehicle, 85.7 ± 9.2 s; CRS/vehicle, 129.5 ± 8.5; CRS/D4, 95.2 ± 8.1 s) (Fig. 4K). In the SPT, CRS led to a reduction in the sucrose preference. D4 treatment also significantly increased the sucrose preference of CRS-exposed mice (Control/vehicle, 69.9 ± 6.9%; CRS/vehicle, 51.1 ± 6.2%; CRS/D4, 67.0 ± 4.5%) (Fig. 4L).

Next, we compared depressive-like behavior using the depressive-like z scores. Oral administration of D4 in a different treatment paradigm did not affect depressive-like z scores in the unstressed control mice (Vehicle, 0.00 ± 0.12; D4, − 0.22 ± 0.15) (Fig. 4G). Indeed, mice exposed to CRS exhibited significantly higher depressive-like z scores than the control/vehicle group. The CRS-induced depressive-like phenotype in mice was again ameliorated by D4 (Control/vehicle, 0.00 ± 0.14; CRS/vehicle, 1.02 ± 0.15; CRS/D4, 0.22 ± 0.13) (Fig. 4N). Given that two doses of D4 were administered before the beneficial effects in TST were observed, the earliest positive effects exerted by D4 were approximately two days. These results could consolidate the antidepressant-like effects of D4 and demonstrate its fast-acting potential using a different mouse model of depression.

### D4 reduces CRS-mediated hemichannel activity and reactive glia

**Fig. 5:**
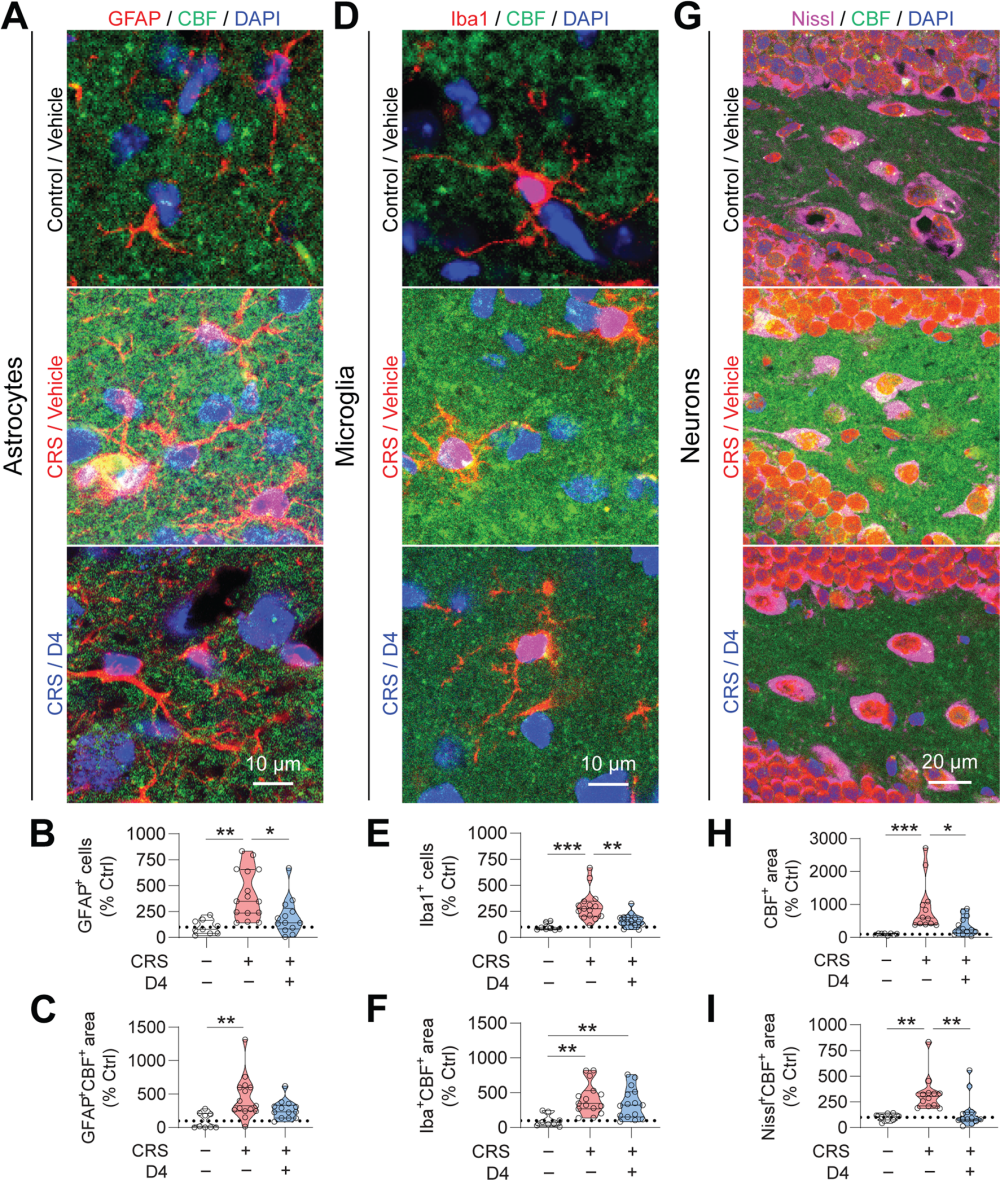
D4 decreases CRS-mediated hemichannel activity and glial activation. **A** Representative images showing astrocyte (GFAP) and CBF in the DG from the CRS-exposed mice. GFAP, red; CBF, green; DAPI, blue. Scale bar, 10 µm. **B** Quantification of GFAP^+^ astrocytes and (**C**) GFAP^+^CBF^+^ area in the stained DG sections. Control/vehicle, n = 9 DG sections from 3 mice; CRS/vehicle, n = 15 DG sections from 5 mice; CRS/D4, n = 13 DG sections from 5 mice. **D** Representative images showing microglia (Iba1) and CBF in the DG from the CRS-exposed mice. Iba1, red; CBF, green; DAPI, blue. Scale bar, 10 µm. **E** Quantification of Iba1^+^ microglia and **F** Iba1^+^CBF^+^ area in the stained DG sections. Control/vehicle, n = 8 DG sections from 4 mice; CRS/vehicle, n = 15 DG sections from 5 mice; CRS/D4, n = 15 DG sections from 5 mice. **G** Representative images showing CBF uptake in the hippocampal DG from the CRS-exposed mice treated with vehicle (Left) and D4 (Right). Nissl, magenta; CBF, green; DAPI, blue. Scale bar, 20 µm. **H** Quantification of CBF^+^ area and **I** Nissl^+^CBF^+^ area in the hippocampal DG sections. Control/vehicle, n = 8 DG sections from 3 mice; CRS/vehicle, n = 12 DG sections from 5 mice; CRS/D4, n = 13 DG sections from 5 mice. The open circle represents the value from each section. Control/vehicle (Ctrl), white; CRS/vehicle, red; CRS/D4, blue. Kruskal–Wallis test with *Bonferroni* correction (**B**, **C**, **E**, **H**, **I**). One-way ANOVA followed by LSD post hoc test (**F**). *p < 0.05, **p < 0.01, ***p < 0.001

Chronic stress-induced changes in glial cell density and functions have been proposed to contribute to mood disorders, including depression [58]. Previous work reported that restraint stress increases glial activation and hemichannel activity in the hippocampus, which may be associated with behavioral deficits induced by chronic stress [39]. In the LPS-induced mouse model of depression, we found that D4 decreases hemichannel-mediated dye uptake (Fig. 1) and the number of reactive astrocytes in LPS-induced depressive-like mice (Fig. 3). To corroborate these results in the CRS-induced mouse model of depression, we measured hemichannel activity in the hippocampal DG from mice treated with either the vehicle or D4 by using CBF uptake. Consistent with results from the LPS model, treatments with D4 induced significant reductions in the number of GFAP^+^ astrocytes (Control/vehicle, 100.0 ± 23.0%; CRS/vehicle, 418.6 ± 62.0%; CRS/D4, 199.7 ± 48.8%), as well as a decrease in the CBF uptake in GFAP^+^ astrocytes (Control/vehicle, 100.0 ± 39.0%; CRS/vehicle, 444.5 ± 81.7%; CRS/D4, 267.6 ± 38.8) in the hippocampal DG (Fig. 5A–C). Compared to vehicle-treated unstressed control mice, CRS led to an increase in the DG Iba1^+^ microglia. CRS mice treated with D4 also showed a significant decrease in the number of Iba1^+^ microglia (Control/vehicle, 100.0 ± 39.0%; CRS/vehicle, 303.9 ± 37.6%; CRS/D4, 160.3 ± 16.5), and a tendency to reduce CBF uptake in the Iba1^+^ microglia (Control/vehicle, 100.0 ± 31.4%; CRS/vehicle, 417.2 ± 58.6%; CRS/D4, 347.8 ± 57.6) in the hippocampal DG (Fig. 5D–F). In the CRS-exposed mice, D4 also reduced CBF uptake (Control/vehicle, 100.0 ± 31.4%; CRS/vehicle, 917.6 ± 221.1%; CRS/D4, 341.4 ± 77.4) and dye uptake in Nissl^+^ neurons (Control/vehicle, 100.0 ± 12.1%; CRS/vehicle, 329.1 ± 51.1%; CRS/D4, 147.1 ± 43.1) in the hippocampal DG from mice subjected to CRS (Fig. 5G–I). Thus, these results suggest that D4 can suppress dye uptake through hemichannel and glial activation induced by CRS, indicating potential mechanisms that contribute to antidepressant-like behavioral outcomes of D4.

### D4 restores neuronal activity in CRS-induced depressive-like mice

Alterations in neural activity and brain network connectivity are consistently associated with depression [59–62]. Glial cells are important for supporting the functions of neurons [63, 64]. In addition, astroglial Cx hemichannels have been shown to regulate behavior by modulating neuronal activity [65, 66]. Therefore, we asked whether the antidepressant-like effects of hemichannel blockade with D4 could be accompanied by changes in neural activity.

**Fig. 6:**
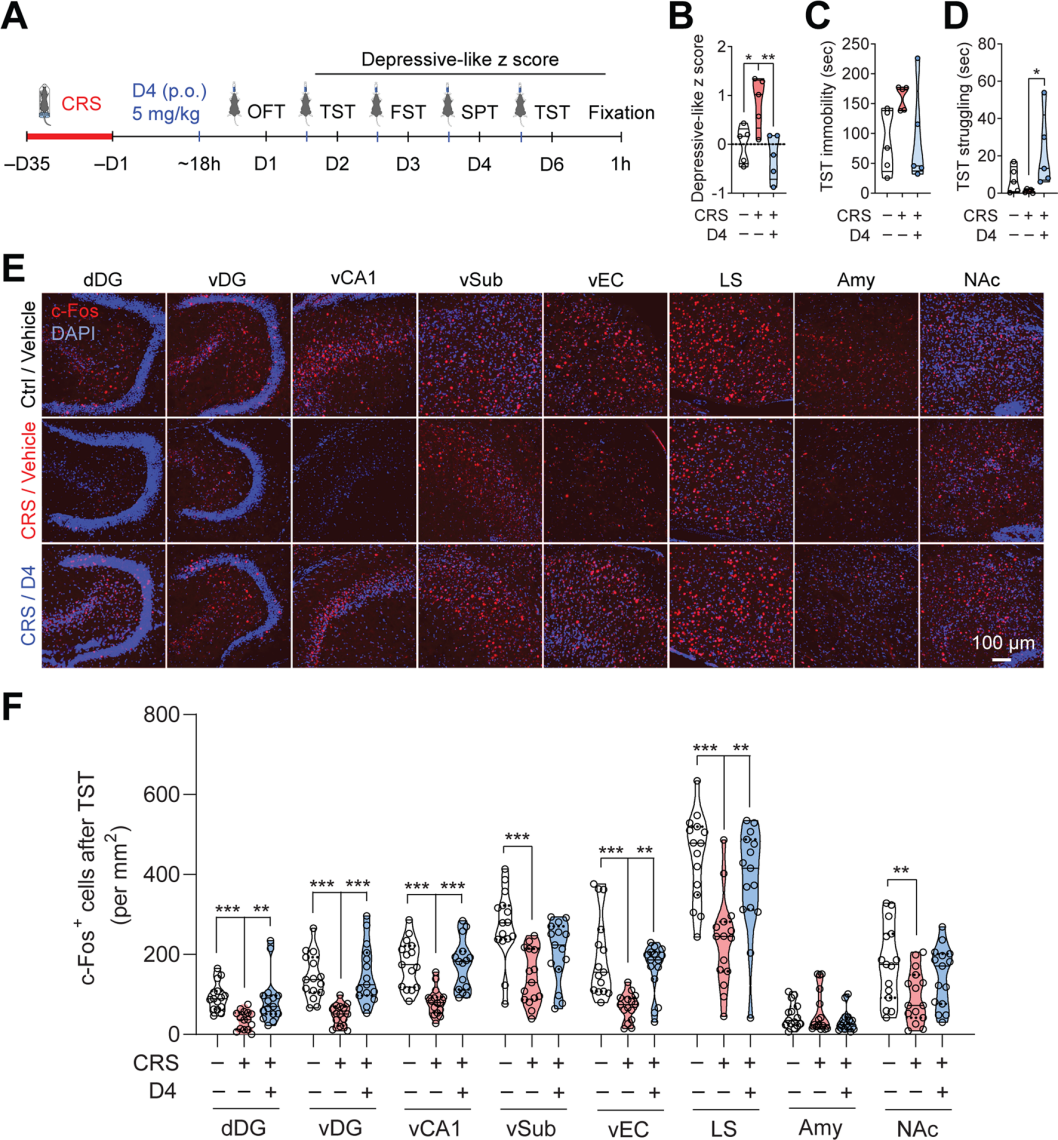
D4 rescues hypofunction of depression-related brain regions in the CRS-induced depressive-like mice. **A** Schematic of the design of CRS, drug treatment, behavioral testing, and c-Fos immunostaining after tail suspension test (TST). **B** Depressive-like z score of mice used for the TST and c-Fos experiments. **C** Time immobile and struggling (**D**) in the TST. The filled dot shows the value of each mouse. Ctrl/vehicle, white; CRS/vehicle, red; CRS/D4, blue. n = 5 mice for all groups. Data are mean ± s.e.m. One-way ANOVA followed by LSD post hoc test (**B**). Kruskal–Wallis test with *Bonferroni* correction (**C**, **D**). **E** Immunostaining for c-Fos expression (red) in the depression-associated brain regions of ctrl/vehicle, CRS/vehicle, and CRS/D4 treated mice (from top to bottom). The nuclei were stained with DAPI (blue). Scale bar, 100 µm. dDG: dorsal dentate gyrus; vDG: ventral dentate gyrus; vCA1: ventral cornu ammonis 1; vSub: ventral subiculum; vEC: ventral entorhinal cortex; LS: lateral septum; Amy: amygdala; NAc: nucleus accumbens. **F** Violin plot of c-Fos^+^ cells in each region after the TST. Ctrl/vehicle, white; CRS/vehicle, red; CRS/D4, blue. The open circle indicates the value from each section. n = 15 sections from 5 mice for all groups. One-way ANOVA followed by LSD post hoc test (LS and NAc). Kruskal–Wallis test with *Bonferroni* correction (dDG, vDG, vCA1, vSub, EC, and Amy). *p < 0.05, **p < 0.01, ***p < 0.001

After CRS and the battery of four behavioral tests (OFT, TST, FST, and SPT), mice were subjected to one last TST, which acted as an acute stressor, and were sacrificed 1 h later to evaluate neuronal activation (Fig. 6A). Similar to previous results (Fig. 4), CRS increased depressive-like z scores in mice, and D4 treatment (5 mg/kg, p.o.) reduced mouse depressive-like z scores (Depressive-like z score, ctrl/vehicle, 0.00 ± 0.17; CRS/vehicle, 0.86 ± 0.24; CRS/D4, − 0.26 ± 0.21) (Fig. 6B). In the TST, D4 (5 mg/kg, p.o.) decreased immobility time, and significantly increased struggling time compared to CRS/vehicle group (TST immobility, ctrl/vehicle, 85.0 ± 23.3 s; CRS/vehicle, 161.1 ± 9.1 s; CRS/D4, 92.3 ± 36.5 s. TST struggling, ctrl/vehicle, 7.2 ± 3.1 s; CRS/vehicle, 1.1 ± 0.4 s; CRS/D4, 22.3 ± 9.0 s) (Fig. 6C, D). This suggests that D4 reduces depressive-like behaviors in the CRS-exposed mice.

Subsequently, we assessed neuronal activation using c-Fos immunostaining following acute exposure to stress (TST). In mice subjected to CRS, there were significantly fewer c-Fos^+^ cells in several depression-related brain regions, including the dorsal and ventral hippocampus (dDG, vDG, vCA1, vSub), ventral entorhinal cortex, lateral septum, and nucleus accumbens (NAc). CRS-exposed mice treated with D4 (5 mg/kg, p.o.) exhibited increases in the number of c-Fos^+^ cells in the hippocampus (dDG, vDG, vCA1), entorhinal cortex, and lateral septum. No significant change in the number of c-Fos^+^ cells was found in the amygdala (Fig. 6E, F). These data suggest that D4 may improve behavioral deficits in depressive-like mice by restoring neural activity in various brain regions.

## Discussion

Hemichannels are important for regulating cellular communication and maintaining homeostasis [8]. Uncontrolled hemichannel activation has been proposed to disrupt neuron-glia communication, promote inflammation, and lead to cell damage in various brain diseases [7]. Recent evidence suggests that elevated hemichannel activity is associated with pathological changes in depression [67]. Therefore, inhibiting hemichannel activity may provide therapeutic benefits, as previously suggested [67–69].

Accordingly, in the present study, we demonstrated the rapid, antidepressant-like effects of D4, which reduced depressive-like symptoms in mice exposed to repeated systemic LPS challenges or CRS. We also showed that blocking hemichannels with D4 inhibits stress-mediated reactive astrogliosis and induces a brain-wide restoration of neural activity in the depressive-like mice subjected to CRS, which may contribute to the behavioral effects of D4. Together, our findings indicate that Cx hemichannels can constitute effective therapeutic targets for depression, and that D4 can be a new molecule mediating this therapeutic effect.

The pathogenesis of depression is highly heterogeneous and is associated with intricate interplays across multiple neurotransmitter systems, neuroimmune systems, neural circuity, and brain networks [70–72]. Considering that neuroinflammatory responses are likely to affect the ontogeny of the central nervous system [73], outcomes under these conditions could be irreversible, and could generate a radical difference compared to the effects of neuroinflammation that only occurs in adulthood. These differences limit current treatment outcomes and dramatically impede the rational design of successful therapies.

Cx hemichannels have been suggested as potential targets for depression [24, 67, 74]. Several agents, including small molecules, antibodies, and peptides, are available for inhibiting Cx hemichannels, with most studies carried out in vitro [18]. However, the behavioral benefits of hemichannel inhibition in depression in vivo remain elusive. In the present study, using two well-established mouse models of depression, we provide substantive evidence for the first time that D4 treatment can improve stress-induced depressive-like symptoms in adult mice. In addition, adult mice that received either acute or chronic applications of D4 did not show evident side effects. The latter could suggest that D4 is a relatively safe Cx hemichannel inhibitor with therapeutic effects. The antidepressant-like potential of D4 still needs to be substantiated for future translational research and clinical studies. Future work is also required to determine its bioavailability and druggability.

It has been shown that hemichannel activity can increase due to inflammation and chronic stress in adult animals [39, 67]. One of the prevailing hypotheses is that abnormal hemichannel activity may amplify detrimental effects on several cellular processes and promote the pathogenesis of a range of neurological and neurodegenerative diseases [10, 16, 43, 75, 76]. For example, in animal models of brain injury, such as ischemic stroke, hemichannels are activated in response to injury and pro-inflammatory cytokines. Dysregulated hemichannel activity can facilitate excitotoxicity due to the excessive release of gliotransmitters and may lead to neuronal death [77]. During epileptogenesis, seizures increase glial hemichannel activity [16, 78]. In turn, such heightened hemichannel activity promotes seizure generation and propagation through neuroinflammation and neuronal hyperexcitability [15, 79]. In animal studies, currently available hemichannel inhibitors have been used to control diseases like ischemia and epilepsy [12, 15]. Consistent with a previous study [41], we found that a single high-dose LPS injection increases glial hemichannel activity in the hippocampus. In addition, we found that repeated low doses of LPS challenges can increase Cx hemichannel activity in the hippocampal DG. Blocking hemichannels with D4 also prevented LPS-mediated reactive astrogliosis, probably due to reduced hemichannel activity in astrocytes, as evidenced by decreased CBF dye uptake. Thus, decreases in Cx hemichannel activity may contribute to antidepressant-like behavioral effects exerted by D4 in the LPS-induced depression model. These findings are consistent with the hypothesis that inhibition of Cx hemichannels by small molecules or other agents can improve depressive-like behaviors.

Our finding that blocking Cx hemichannels with D4 restored neuronal activity in CRS-exposed depressive-like mice provides another mechanistic insight into how hemichannel inhibition can contribute to behavioral improvement. Reduced neuronal activity in several regions of the limbic system has been identified in the etiology of depression [80]. Notably, post-mortem and neuroimaging studies have confirmed that the hippocampus is hypoactive in patients with depression [81, 82]. Antidepressant treatments can reverse depression-associated structural and functional changes in the hippocampus [83–85].

Similar to previous findings, we found that mice acutely exposed to CRS display significant reductions in neuronal activity in the hippocampus [86] and several other limbic regions, including the entorhinal cortex and the lateral septum. In contrast, D4 treatment reduces depressive-like behavior in the TST and enhances neural activation, as evidenced by the recovery of c-Fos^+^ cells in these regions. Although our dye uptake and glial density assays were restricted to the hippocampal DG regions, findings from our c-Fos screening results raise the possibility that D4 could impact the hemichannel and neural activity in multiple sites across the brain after systemic application via oral administration. Evidence for this possibility is supported by our recent study showing that oral treatment of D4 with optimized doses is sufficient to reduce seizure-induced glial cell density and hemichannel activity in different subfields of the hippocampus and anterior piriform cortex [16]. Data from clinical and preclinical studies suggest that the route of antidepressant administration may affect therapeutic efficacy and experience, especially for the novel rapid-acting antidepressant such as ketamine [6, 85]. Thus, specific mechanisms by which CRS reduces neuronal activation and D4 regulates neural activity remain to be studied with more sophisticated and precise targeted drug delivery strategies.

Cx43 is predominantly expressed in astrocytes in the adult mouse brain. Resting microglia rarely express Cx43, but its expression level can be increased in activated microglia [75, 87, 88]. Previously, it has been shown that D4 inhibits Cx43 hemichannels but not gap junction channels [29]. In this study, we showed that D4 reduces LPS-induced CBF uptake in astrocytes without affecting dye uptake in microglia. D4-induced reduction in astroglia-mediated hemichannel activity could indirectly reduce pannexin1 hemichannel activity in neurons, as observed under neuroinflammation triggered by a neurotoxic beta-amyloid peptide [89]. Based on existing studies suggesting that hemichannels can regulate gliotransmitter release, synaptic transmission, and neuronal firing [90–94], we speculate that the behavioral effect of D4 is likely mediated by the initial inhibition of astrocytic Cx43 hemichannel activity, which subsequently affects neuron–glia interaction and neuronal function. A limitation of our current study was the lack of detailed comparisons between sexes in their sensitivity to chronic stressors and compound D4. Sex could be a factor that affects the prognosis of depression and the treatment response of antidepressant interventions [2, 5]. It will be interesting to test whether D4 has a sex-specific effect on changes in hemichannel and behavioral activity induced by depressogenic factors. Given that Cx hemichannels can actively regulate neural activity and behavioral states via a cornucopia of cellular processes [64, 65], future studies are needed to elucidate the mechanisms of antidepressant-like behavioral effects of D4.

## Conclusion

In conclusion, we have shown that a recently discovered hemichannel inhibitor, namely the small organic molecule D4, can exert antidepressant-like effects in mice subjected to repeated systemic LPS challenges or CRS. These behavioral benefits of D4 are accompanied by the blockade of hemichannel activity reduced inflammatory response, and a brain-wide restoration of neural activity in depressive-like mice. Our findings support the hypothesis that the hemichannel inhibitor is a promising pharmacophore for future antidepressant development focusing on glial cells. A better understanding of D4’s cellular mechanisms may help to identify its therapeutic potential and promote clinical uses of hemichannel inhibitors in mood disorders.

### Supplementary Information

**Additional file 1: Fig. S1.** D4 does not inhibit TRPV2 channels. **A–D** HeLa parental cells (Cx45^−/−^) were transfected with pIRES2-EGFP-hTRPV2 vector (0.5 µg/10^5^ cells) and 24 h later channel activity was assessed through calcium imaging with Fura-2. TRPV2 channel was opened with 50 µM 2-Aminoethoxydiphenyl borate (2-APB), an effect that was maintained even after 3 washes (**A**). When La^3+^ was added to TRPV2 channels already open with 2-APB, Ca^2+^ influx was blocked (**B**), whereas 500 nM D4 did not affect TRPV2 activity (**C**). D4 pre-incubation did not prevent the opening of TRPV2 induced by 2-APB (**D**). 10 µM A23187 ionophore was used as a positive control for Ca^2+^ influx. Representative graphs of at least 3 evaluations for each experiment. Each point represents the average of 20–30 cells recorded in each condition.

**Additional file 2: Fig. S2.** D4 does not inhibit P2X_7_R channels. **A–C** HeLa parental cells (Cx45^−/−^) were transfected with pIRES2-EGFP-hP2X_7_R vector (0.5 µg/10^5^ cells) and 24 h later channel activity was assessed through calcium imaging with Fura-2. In all assays, P2X_7_R channel was opened with 2 mM adenosine triphosphate (ATP) and then the effect of 100 nM (**A**), 500 nM (**B**) and 1 µM (**C**) of D4 was evaluated. Application of D4 (100 nM, 500 nM, or 1 µM) did not affect the opening of P2X_7_R induced by ATP, whereas 20 µM A740003 inhibited the ATP-mediated P2X_7_R channel opening in all experiments. Representative graphs of at least 2 evaluations for each experiment. Each point represents the average of 20–30 cells recorded in each condition.

**Additional file 3: Fig. S3.** D4 does not inhibit CALHM1 channels. **A** HeLa parental cells (Cx45^−/−^) were transfected with human GFP-tagged CALHM1 vector (origene cat#: RG206902, 0.5 µg/10^5^ cells) and 24 h later channel activity was assessed by DAPI uptake. **B** DAPI uptake rates calculated from values as shown in (**A**), considering basal segment (0–5 min), stimulation with divalent cation-free solution (DCFS) (5–10 min) and the effect of ruthenium red (RR, 20 µM) or 0.5 µM of D4 (10–15 min). The slope of dye uptake within the last minute in each condition was quantified (basal, 4–5 min; DCFS, 9–10 min; RR or D4, 14–15 min). RR but not D4 reduced the opening of CALHM1 induced by DCFS. Each point represents the average of 20–30 cells recorded in each condition. n = 6. Data are mean ± s.e.m. One-way ANOVA followed by LSD post hoc test. *p < 0.05, **p < 0.01, ***p < 0.001. **C** HeLa cells stably expressing Cx43 were used as a positive control for D4 (0.5 µM) inhibition. n = 3.

**Additional file 4: Fig. S4.** D4 mitigates inflammatory responses induced by LPS. **A** Timeline of LPS injection and sample collection. **B** ELISA analysis of plasma (left) and hippocampal (right) levels of pro-inflammatory cytokine IL-1β in the saline/vehicle control, LPS/vehicle, and LPS/D4 mice. n = 3 mice per group. The filled dot indicates the value of each mouse. Data are mean ± s.e.m. One-way ANOVA followed by LSD post hoc test. **p < 0.01, ***p < 0.001.

## Data Availability

All data presented in this study are available from the corresponding author upon reasonable request.
