# Supplementary figures and images for: The connexin hemichannel inhibitor D4 produces rapid antidepressant-like effects in mice
Source: J Neuroinflammation. 2023 Aug 20;20:191. doi: 10.1186/s12974-023-02873-z (PMC10440914; doi:10.1186/s12974-023-02873-z)

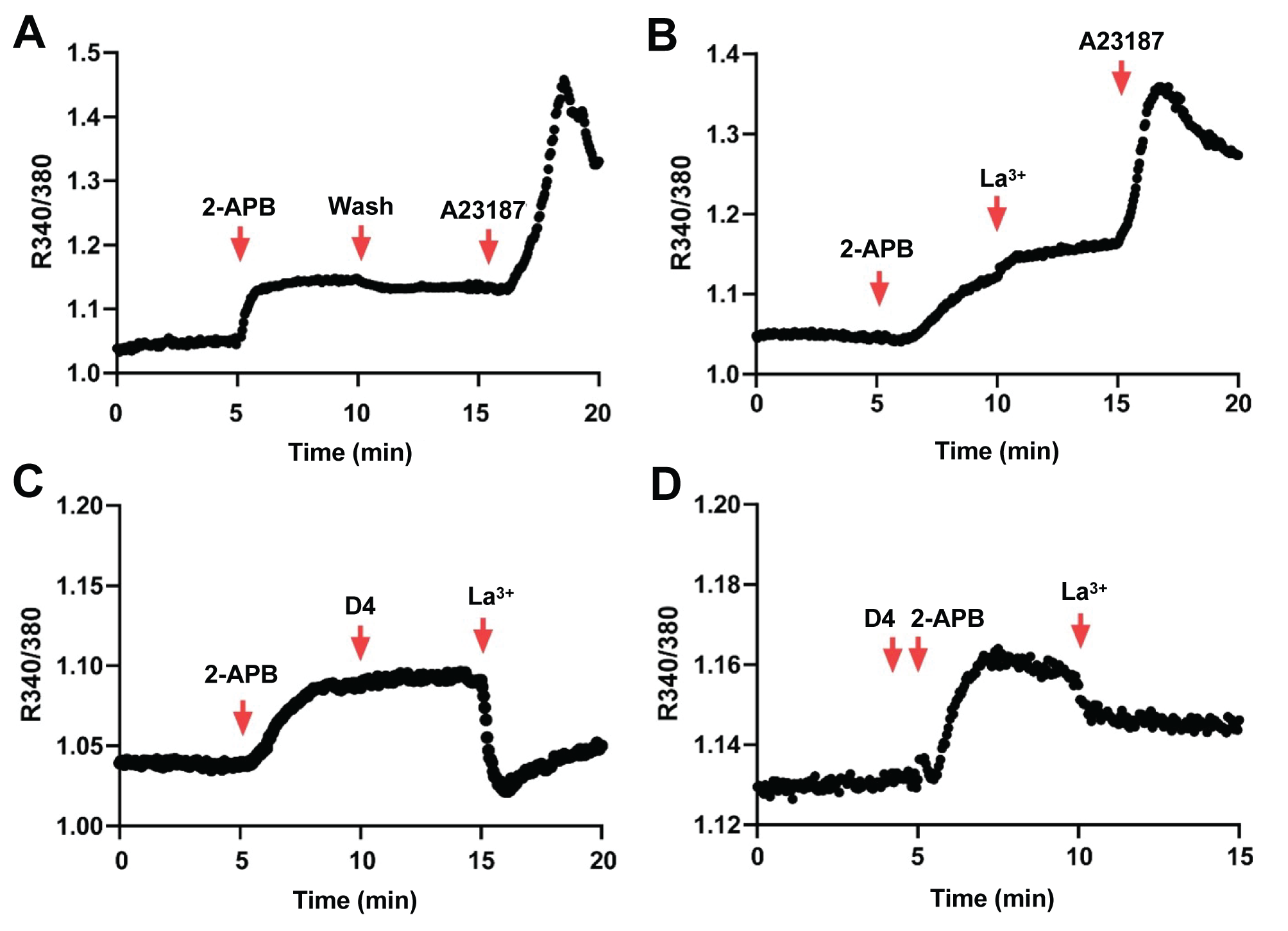

Supplement: Supplementary file 1 — Additional file 1: Fig. S1. D4 does not inhibit TRPV2 channels. A–D HeLa parental cells (Cx45−/−) were transfected with pIRES2-EGFP-hTRPV2 vector (0.5 µg/105 cells) and 24 h later channel activity was assessed through calcium imaging with Fura-2. TRPV2 channel was opened with 50 µM 2-Aminoethoxydiphenyl borate (2-APB), an effect that was maintained even after 3 washes (A). When La3+ was added to TRPV2 channels already open with 2-APB, Ca2+ influx was blocked (B), whereas 500 nM D4 did not affect TRPV2 activity (C). D4 pre-incubation did not prevent the opening of TRPV2 induced by 2-APB (D). 10 µM A23187 ionophore was used as a positive control for Ca2+ influx. Representative graphs of at least 3 evaluations for each experiment. Each point represents the average of 20–30 cells recorded in each condition. [file 12974_2023_2873_MOESM1_ESM.tif]

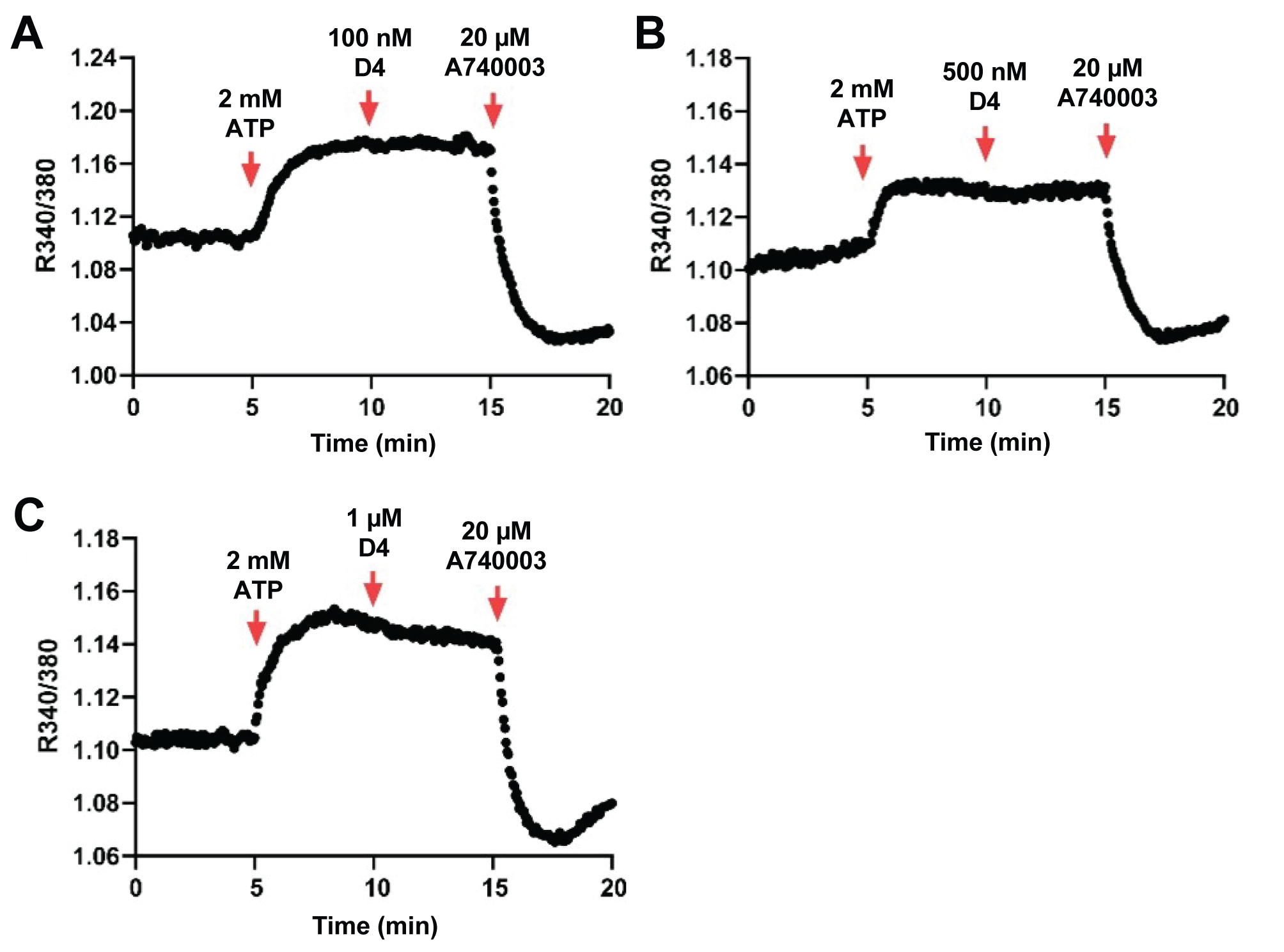

Supplement: Supplementary file 2 — Additional file 2: Fig. S2. D4 does not inhibit P2X7R channels. A–C HeLa parental cells (Cx45−/−) were transfected with pIRES2-EGFP-hP2X7R vector (0.5 µg/105 cells) and 24 h later channel activity was assessed through calcium imaging with Fura-2. In all assays, P2X7R channel was opened with 2 mM adenosine triphosphate (ATP) and then the effect of 100 nM (A), 500 nM (B) and 1 µM (C) of D4 was evaluated. Application of D4 (100 nM, 500 nM, or 1 µM) did not affect the opening of P2X7R induced by ATP, whereas 20 µM A740003 inhibited the ATP-mediated P2X7R channel opening in all experiments. Representative graphs of at least 2 evaluations for each experiment. Each point represents the average of 20–30 cells recorded in each condition. [file 12974_2023_2873_MOESM2_ESM.tif]

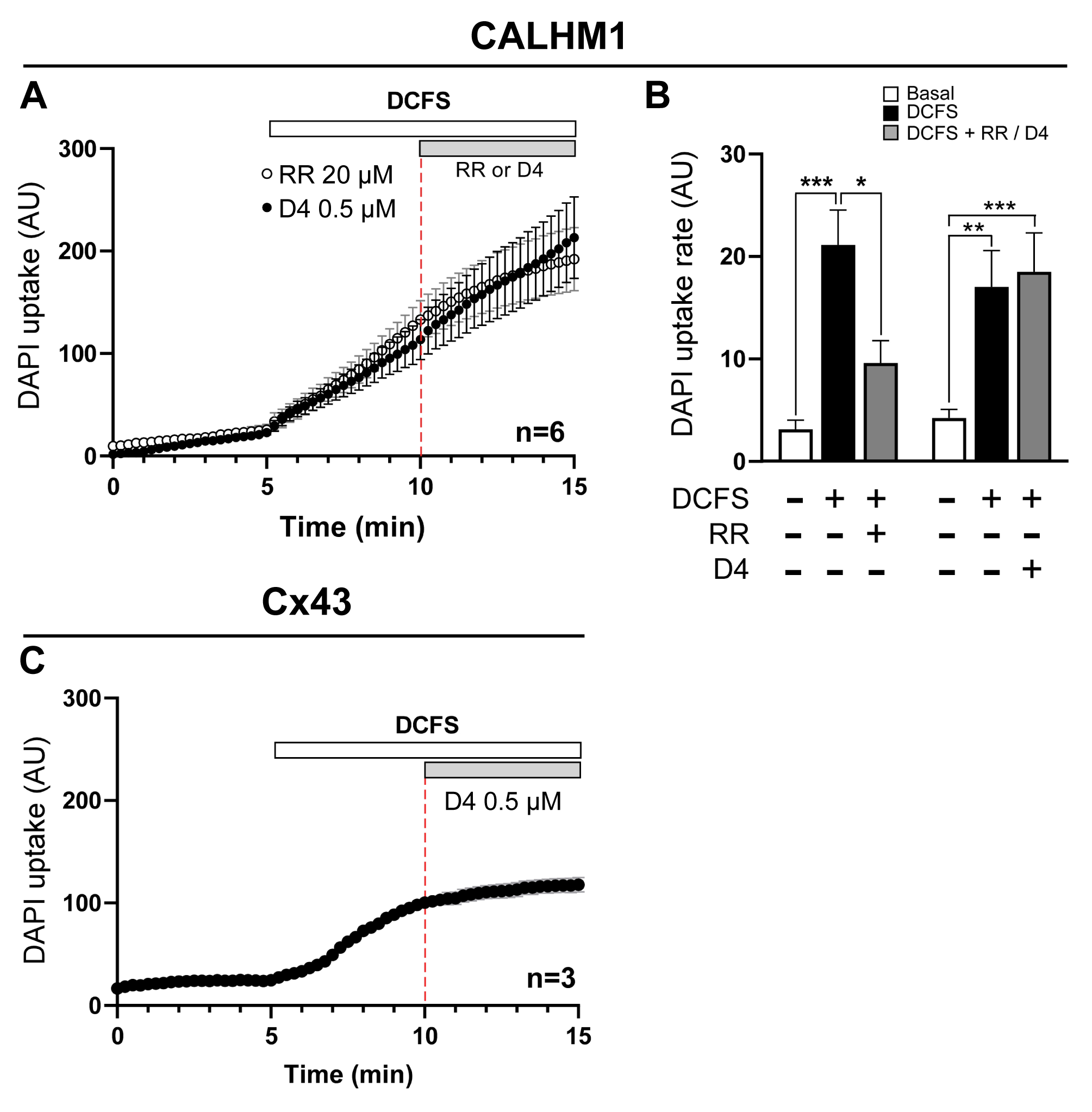

Supplement: Supplementary file 3 — Additional file 3: Fig. S3. D4 does not inhibit CALHM1 channels. A HeLa parental cells (Cx45−/−) were transfected with human GFP-tagged CALHM1 vector (origene cat#: RG206902, 0.5 µg/105 cells) and 24 h later channel activity was assessed by DAPI uptake. B DAPI uptake rates calculated from values as shown in (A), considering basal segment (0–5 min), stimulation with divalent cation-free solution (DCFS) (5–10 min) and the effect of ruthenium red (RR, 20 µM) or 0.5 µM of D4 (10–15 min). The slope of dye uptake within the last minute in each condition was quantified (basal, 4–5 min; DCFS, 9–10 min; RR or D4, 14–15 min). RR but not D4 reduced the opening of CALHM1 induced by DCFS. Each point represents the average of 20–30 cells recorded in each condition. n = 6. Data are mean ± s.e.m. One-way ANOVA followed by LSD post hoc test. *p < 0.05, **p < 0.01, ***p < 0.001. C HeLa cells stably expressing Cx43 were used as a positive control for D4 (0.5 µM) inhibition. n = 3. [file 12974_2023_2873_MOESM3_ESM.tif]

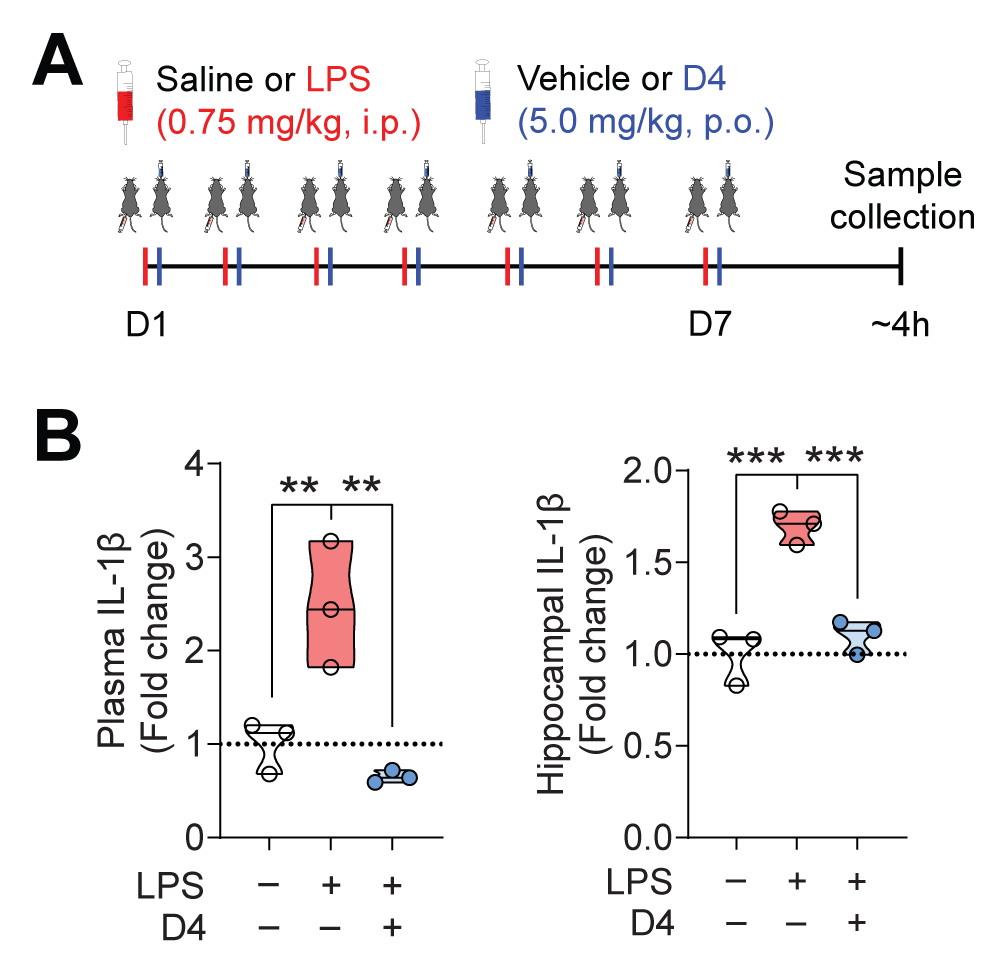

Supplement: Supplementary file 4 — Additional file 4: Fig. S4. D4 mitigates inflammatory responses induced by LPS. A Timeline of LPS injection and sample collection. B ELISA analysis of plasma (left) and hippocampal (right) levels of pro-inflammatory cytokine IL-1β in the saline/vehicle control, LPS/vehicle, and LPS/D4 mice. n = 3 mice per group. The filled dot indicates the value of each mouse. Data are mean ± s.e.m. One-way ANOVA followed by LSD post hoc test. **p < 0.01, ***p < 0.001. [file 12974_2023_2873_MOESM4_ESM.tif]
